# Unraveling Adhesion Strength between Gas Hydrate and Solid Surfaces

**DOI:** 10.1021/acs.langmuir.1c02315

**Published:** 2021-11-16

**Authors:** Rui Ma, Feng Wang, Yuanhao Chang, Senbo Xiao, Niall J. English, Jianying He, Zhiliang Zhang

**Affiliations:** † NTNU Nanomechanical Lab, Department of Structural Engineering, Norwegian University of Science and Technology (NTNU), Trondheim 7491, Norway; ‡ School of Chemical and Bioprocess Engineering, University College Dublin, Belfield Dublin 4, Ireland

## Abstract

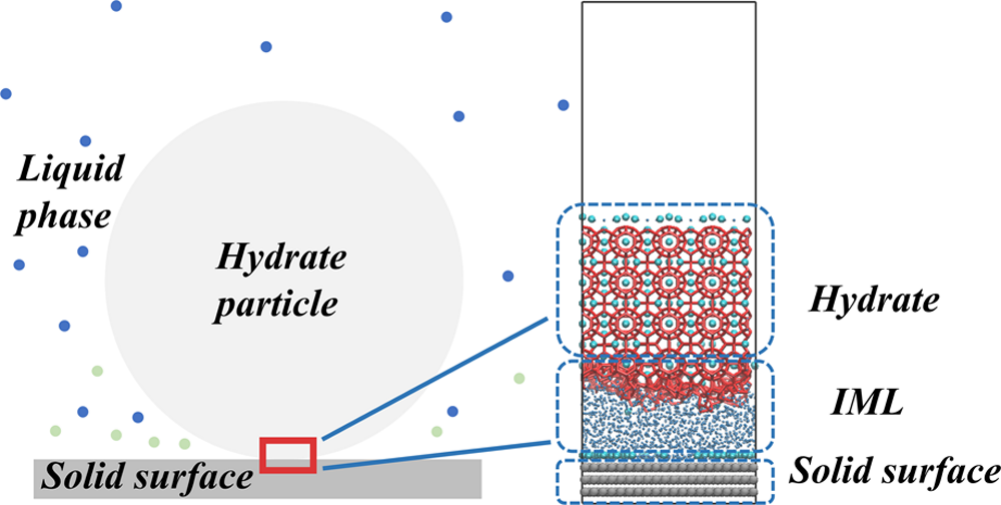

Natural gas hydrate is a promising future energy source, but it also poses a huge threat to oil and gas production due to its ability to deposit within and block pipelines. Understanding the atomistic mechanisms of adhesion between the hydrate and solid surfaces and elucidating its underlying key determining factors can shed light on the fundamentals of novel antihydrate materials design. In this study, large-scale molecular simulations are employed to investigate the hydrate adhesion on solid surfaces, especially with focuses on the atomistic structures of intermediate layer and their influences on the adhesion. The results show that the structure of the intermediate layer formed between hydrate and solid surface is a competitive equilibrium of induced growth from both sides, and is regulated by the content of guest molecules. By comparing the fracture behaviors of the hydrate–solid surface system with different intermediate structures, it is found that both the lattice areal density of water structure and the adsorption of guest molecules on the interface together determine the adhesion strength. Based on the analysis of the adhesion strength distribution, we have also revealed the origins of the drastic difference in adhesion among different water structures such as ice and hydrate. Our simulation indicates that ice-adhesion strength is approximately five times that of lowest hydrate adhesion strength. This finding is surprisingly consistent with the available experimental results.

## Introduction

Natural gas hydrate is considered to be a future energy source. It is conservatively estimated that the energy stored in natural gas-hydrate sediments is about twice that of conventional fossil fuels on the earth.^1,2^ Substantial natural gas hydrates have been found on the continental shelf and in the permafrost regions, and have aroused widespread research interest all over the world.^3−5^ On the other hand, as a metastable phase of water, the hydrate can exist stably under high pressure and low temperature environmental conditions. Its occurrence environment determines that it can nucleate and grow in oil- and gas-extraction and transportation pipelines, especially in the process of deep-water-oil and gas resources. The generation and deposition of hydrates brings the risk of blocking pipelines, which greatly threatens the safety of oil and gas production and transportation.^6−10^

Natural gas hydrate has an ice-like appearance macroscopically and with a cage-like structure on the microscopic level, and some small molecules such as methane, carbon dioxide, hydrogen, and other small hydrocarbons are often trapped in cage cavities formed by water molecules as guests.^3^ Under normal, low-temperature conditions, hexagonal ice (Ih) is always a relatively stable thermodynamic phase than the empty clathrate hydrate structure.^11^ It is almost impossible to form an empty clathrate crystal structure in the pure water phase, which means that guest molecules are indispensable, at least at the beginning of hydrate nucleation.^12^ In fact, high concentrations of solvated guest molecules often trigger rare hydrate-nucleation events. Therefore, hydrate nucleation is often found preferentially near the gas–liquid interface or the gas–liquid–solid contact area.^13−16^ However, based on conclusions drawn from sometimes-conflicting experimental and simulation results, it must be pointed out that it is still a challenge to observe whether hydrates can directly nucleate and grow on the solid surface.^15,17^ Nevertheless, in almost all the studies involving solid surfaces, a hydrate nucleus can eventually form a stable conglutination by forming an intermediate layer (all the following are abbreviated as IML) or connecting with some functional groups at solid surfaces.^18−20^ This microscopic propensity is also the physical basis for the deposition of hydrates. A very dramatic example is that with the growth and agglomeration of the hydrate nucleus, these hydrate particles tend to deposit on the pipeline wall during fluid-flow or shut-down/restart circumstances. The undesired formation of hydrate-plugs creates flow restrictions in the pipeline, often leading to overpressurization and potentially causing catastrophic consequences.

Experimental studies have shown that the hydrate-deposition process initiated by the water layer is a key mechanism causing hydrate-plugs.^21,22^ In addition, there are many experimental results for the adhesion of hydrates on solid surfaces.^23−27^ Although these studies have provided valuable information on hydrate adhesion, due to the limitation of the current experimental resolution, rigorous physical insights into intrinsic adhesion per se are not yet available. The fundamental, open questions regarding the adhesion of hydrates on solid surface can be summarized as follows: (1) How does hydrate establish a connection with solid surface? (2) Is it possible to form an IML? (3) What are the key factors that determine the adhesion of hydrate on solid surfaces? While experimental exploration to these problems is pending, atomistic modeling can scrutinize the microscopic behavior of adhesion, which is surely helpful in tackling some of these pertinent questions, at least initially. The purpose of this research is to explore the formation process of the IML structure when hydrate deposition occurs, and to unravel the key mechanisms that determine hydrate adhesion through tensile testing. This understanding is essential for depicting the process of hydrate deposition and, indeed, in establishing “design rules” in the design and specification of future antihydrate surfaces.

## Computational Methods

### Model Systems

**Figure 1:**
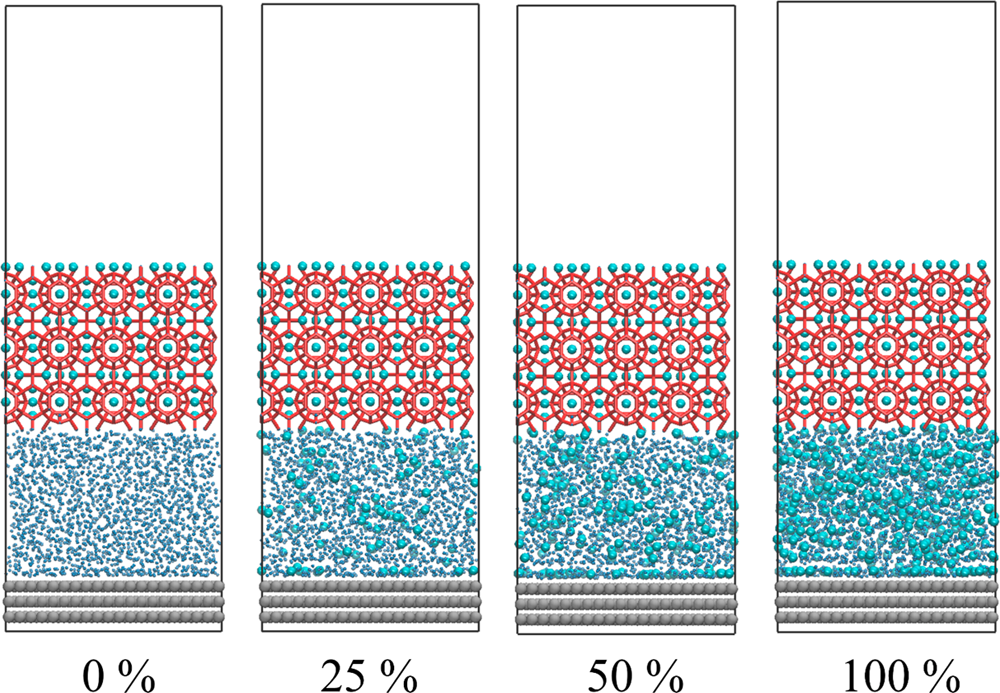
Initial configuration of simulation systems. The solid surface is shown with gray balls, guest molecules with cyan balls, liquid water with blue dots, and hydrate cage with red sticks.

Several sets of hydrate–surface model systems, featuring an amorphous structure containing different concentrations of guest molecules as the IML, have been constructed to simulate the process of adhesion between hydrate and solid surface, as shown in Figure 1. The prebuilt hydrate crystals and solid surfaces are of the same size in all models. The hydrate volume is 4 × 4 × 3 sI hydrate unit cells (2208 water molecules). For the sake of simplicity, the surface is aimed to be smooth to ice and hydrate, meaning having a lattice size smaller than that of ice. The solid surface consists of three layers of smooth hexagonal lattice structure same as graphene. The whole surface is immobilized during simulation. The selection of such a surface can introduce into the system an ability to promote ice nucleation “bottom-up” in addition to the “top-down” ability of hydrates to induce IML growth.^28^ This arrangement can generate the extreme structure of the IML in the competitive growth of ice and hydrate, and, more significantly, shows the difference in adhesion caused by the change in the structure of the IML. The difference of these models is reflected in the different content of guest molecules in the IML between the hydrate crystal and solid surface. Changing the concentration of different guest molecules is used to simulate the change of the IML structure when there are different amounts of dissolved guests in the surrounding liquid phase. In this set of models, the IML containing the sI hydrate guest-water ratio (2300 water molecules and 400 guests) is marked as 100%, and the number of guest molecules contained in the rest of the model system was reduced to 50%, 25%, and 0%. All IML contains the same number of water molecules.

### Force-Fields and Parameters

The mW model is employed to describe the interaction between water molecules.^29^ Each water molecule is represented by a single particle capable of forming a tetrahedral “hydrogen-bond” structure, and the interaction between water molecules is determined by the Stillinger–Weber (SW) potential.^30^ Since this model has a lower energy barrier to be crossed during phase transitions, it is easier to obtain an extreme IML structure, namely ordered structures such as ice or hydrate. This model thus has been widely used in the study of ice and hydrate.^28,31−36^ The “M” particle used for the guest molecule is also a monatomic model represented by the S–W two body interaction, which can represent methane or other similar small molecules.^31,32^ The solid surface is immobilized during the whole simulation process as mentioned previously, and there is no interaction between its internal atoms. All force-field parameters involved in this study can be found tabulated in previous work.^37^

### Simulation Settings

MD simulations were performed using LAMMPS.^38^ A quasi-statical minimization was executed, and all the obtained local minimum-energy configurations underwent further relaxation with a simulation time of 1 ns at 270 K after fixing the hydrate and solid surface. Then, the spatial restriction on the hydrate crystals was lifted, and the system was quenched to a temperature of 210 K for *NVT*-ensemble simulation to realize the IML between hydrate crystals and the solid surface. All simulation boxes have the same size (48.12 Å × 48.12 Å × 140 Å, including the thickness of the vacuum layer). The Newton’s equations of motion were integrated with the velocity-Verlet algorithm with a time step 10 fs,^29^ and all directions of the simulated box used periodic boundary conditions. The Nosé–Hoover algorithm was employed to control the temperature with a relaxation time of 1 ps.^39,40^ The simulation time of all models is generally 100 ns or more depending on the formation of stable IML, and each model had at least five parallel runs undertaken, with Maxwell–Boltzmann velocity randomization of starting structures similar to Figure 1, to ensure statistical reproducibility, and to estimate standard deviation.

## Results and Discussion

### Growth Process of IML

**Figure 2:**
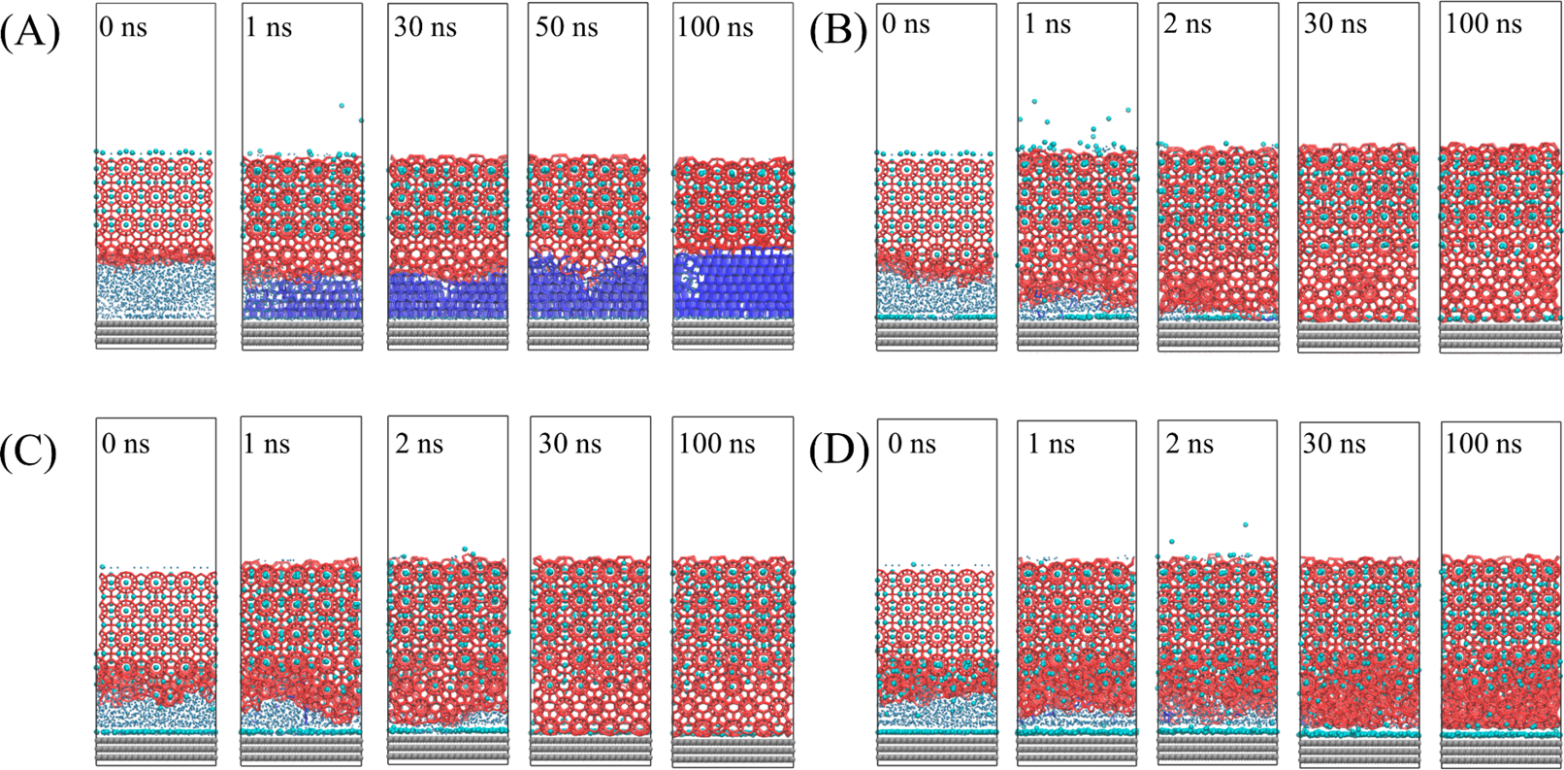
Snapshots of dynamic trajectories for the MD system. (A) With 0% guest molecule content. (B) With 25% guest molecule content. (C) With 50% guest molecule content. (D) With 100% guest molecule content. The solid surface is shown with gray balls, guest molecules with cyan balls, liquid water with blue dots, and ice and hydrate with navy blue and red sticks, respectively.

A topology-based recognition algorithm^41^ and CHILL+ algorithm^42^ were used to trace the growth of hydrate and ice. The total number of water molecules contained in these different phase structures allow for the definition of its size, and this definition is commonly used to indicate the size of ice and hydrate in MD studies.^28,41^ The typical simulation snapshots of the four sets of models have shown in Figure 2, in which the structures that belong to hydrate and ice have been identified and displayed as red and blue sticks, respectively. All of the snapshots marked as “0 ns” are the configurations that are quenched to 210 K after being equilibrated at 270 K. From these configurations, it can be found that some amorphous water molecules in the IML near the hydrate interface already change into the hydrate crystals under the strong induction of the prebuilt hydrate lattice. The number of these newly induced hydrate structures obviously decrease as the concentration of guest molecules decreases. This phenomenon implies that the concentration of solvated guest molecules during the growth process will greatly affect the growth rate of hydrates. It is particularly noteworthy that in the configuration where the IML is a pure water structure, the hydrate crystals induce a layer of empty cage structures. The result is consistent with the research that the growth of hydrate lattice does not need the presence of a guest molecule.^12^ The growth front of the hydrate already clearly continued to advance within a simulation time 1 ns in all the models (Figure 2). In all configurations where the IML contains guest molecules, the guest molecules are captured by the hydrate lattice, and some guest molecules are also adsorbed to the solid surface to form a guest-molecule adsorption layer–effectively, a “nano-bubble”, via the guest-supersaturation driving force for hydro-phobic/-philic phase segregation designed in IML from the outset.^43,44^ Some empty cages appeared during the growth of the hydrate lattice in all models. This is because the hydrogen-bond induction from the hydrate structured water molecules as the host dominates this structural-rearrangement process (reorientation of water molecules’ coordination layers) in this incipient hydrate-growth (as opposed to nucleation) stage.

In addition, another effect of promoting ice nucleation on the solid surface is also seen keenly “at play”. Ice quickly nucleates on the solid surface and grows rapidly in the system marked “0%”, as shown in Figure 2(A), which is similar to the phenomena observed on the surface of single-layer graphene.^28^ There is also noticed that a clear competition between the ice structure that nucleates and grows outward from the solid surface and the hydrate front that grows in the other direction. With the extension of simulation time, the previously generated empty hydrate will rearrange and transform into an ice structure. This phenomenon shows that under current environmental conditions, ice is, unsurprisingly (in view of the van der Waals-Platteeuw statistical-thermodynamics framework^45^), a more stable phase than empty hydrates, which also reflects that guest molecules play a very important role in stabilizing the hydrate structure. In all other models containing guest molecules, the nucleation of ice becomes very rare (there are only two special cases in dozens of simulations, and the snapshots are shown in Supporting Information (SI) Figure S1), especially bearing in mind the high concentration of guest molecules; the nanobubble nature of the guests in the IML after more thorough phase segregation hindered facilitating of ice formation in these selected, rare cases. This phenomenon indicates that the guest molecules adsorbed atop the solid surface may prevent water molecules from forming an ordered structure of ice nucleus.^46^ Therefore, in this type of model, we have only observed an orderly propagation growth of hydrate structure, as shown in Figure 2(B–D). Now, to be sure, there are also obvious differences in the transformed IMLs in between runs, as well as between the various starting configurations. In addition to the random distribution of a large number of empty cage structures in the IML, we also found that the higher concentration of guest molecules is not conducive to the evolution of the hydrogen-bond network structure transforming to a single phase, as shown in Figure 2(D). This observation can be attributed to the hydrophobic effect and steric hindrance, in that a large number of guest molecules, especially with a good deal of supersaturation, will change the original structural orientation of water molecules.^47,48^

However, there is no doubt that the water molecule clusters near these guests in Figure 2(D) must have 5-rings or 6-rings water structures which belong to the substructure of hydrate, so that it can be recognized by our topological algorithms, with the pentamer being a particularly characteristic structural-signature motif of hydrates. These structures are similar to the amorphous hydrate structure, such as the precursor in the nucleation process. The structure is also simulated for a longer time (500 ns) to verify the stability (growth curve and snapshots see SI Figures S3 and S4). These results show that further ordering of the structure is very difficult in the possible time range covered by MD simulation. One of the important reasons may be that due to local deficiency of guest molecules (upon phase segregation and nanobubble formation), a large number of empty cages, have lost the driving force for further compressing the hydration shell, as the previously observed “drainage” process by guest molecules was disabled.^41^

**Figure 3:**
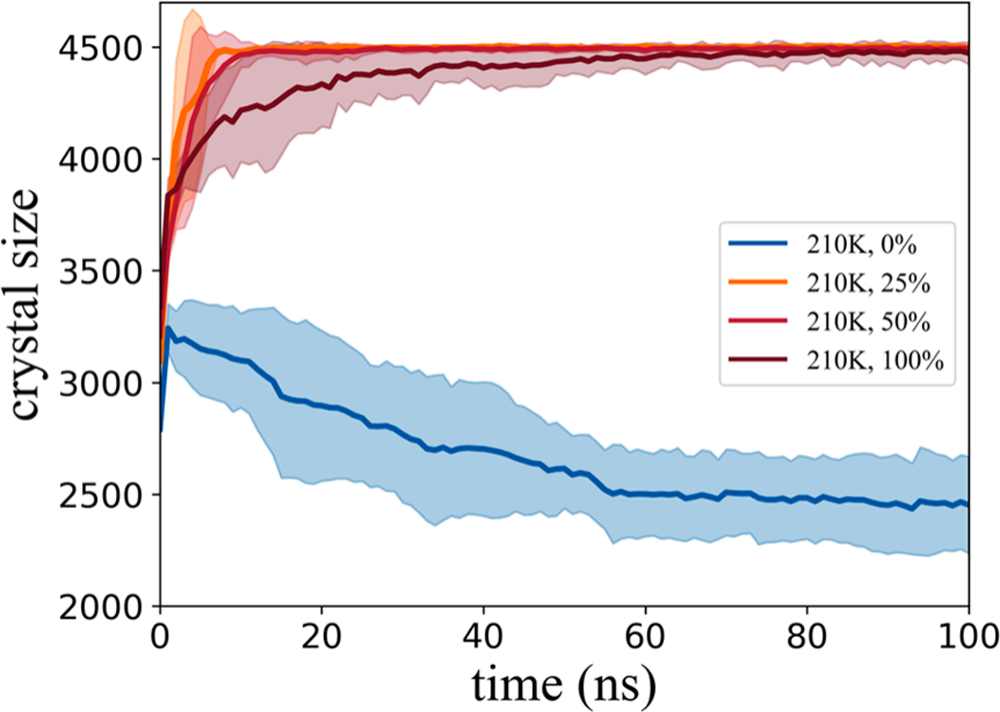
Growth curve of hydrate in all model systems. The average hydrate crystal size in each system is given as solid color lines, with the corresponding standard deviations of five independent runs given as similar faded colors. It should be noted that there is an outlier of ice nucleation in the model set marked “25%”, and its growth curve is shown separately in SI Figure S2.

**Figure 4:**
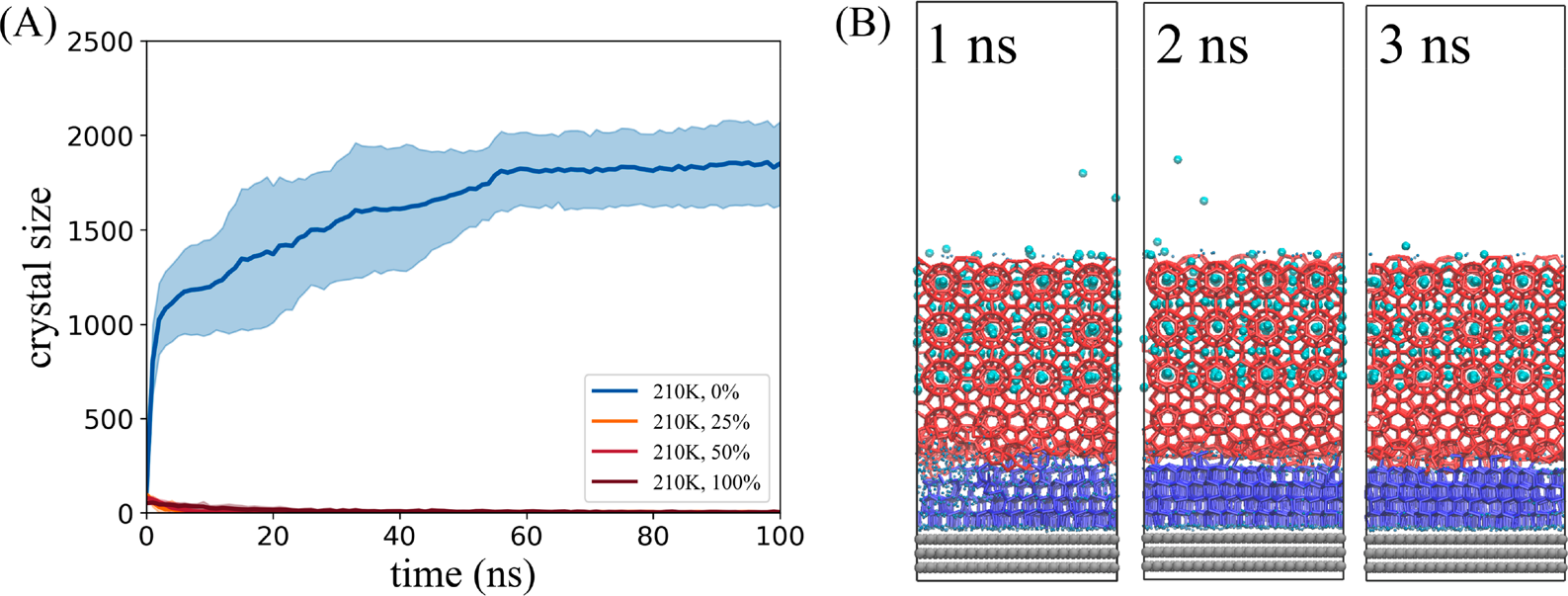
Competitive growth between ice and empty hydrate cages. (A) The average ice crystal size in all model systems is given as solid lines, with the standard deviations of five independent runs shown as similar faded colors. (B) Snapshot of ice and empty hydrate cage growing to forming interface. The solid surface is shown with gray balls, guest molecules with cyan balls, liquid water with blue dots, and ice and hydrate with navy blue and red sticks, respectively.

By counting the number of water molecules contained in the hydrate structures, the growth curve of the hydrate is shown in Figure 3. Obviously, it can be seen that the growth rate of hydrate in all the models in which the IML contains guest molecules is significantly faster than that in the pure water model. This shows that the presence of guest molecules will significantly accelerate the formation of hydrate structures, but a high content of guest molecules seems to reduce the growth rate of hydrates, which also can be attributed to the accumulation of hydrophobic effect and steric hindrance of the locally concentrated guest molecules. In addition, we must point out that when the simulation reaches ∼3 ns, the contact between the growth front of the hydrate structure and the ice structure is also an important reason why the hydrate structure is difficult to continue to grow. It can be seen from the change of the growth curve that after a few nanoseconds of short-term, the hydrate structure begins to decrease significantly. Combined with the ice growth curve (Figure 4(A)), it can be found that the original empty hydrate structure induced by the prebuilt hydrate lattice begins to decrease continuously, and then transforms into an ice structure at the hydrate–ice interface, until the empty hydrate cage is almost completely consumed. After ∼58 ns, the growth of hydrate and ice reached a relatively stable stage, which indicates that ice phase cannot induce hydrate-containing guest molecules to further transform into ice under the current conditions, which again reflects the stabilization effect of guest molecules for hydrate structure.

The rapid increase of the ice growth curve before ∼3 ns is due to the fact that the ice is surrounded by a disordered liquid phase near the solid surface at the initial stage of nucleation, so the ice can quickly nucleate and grow rapidly. After ∼3 ns, due to the exhaustion of the disordered liquid phase, the ice starts to induce the empty hydrate cage transition to ice, and hence leads to a substantial decline in the growth rate. Figure 4(B) shows a series of snapshots of ice and empty hydrate cage growing independently until forming a competing interface.

In summary, the deposition of hydrate particles on a solid surface will induce the formation of an IML, which establishes a stable connection between the hydrate particles and the solid surface. In the model design, the solid surface we selected appropriately stands out its templating ability for the liquid phase in a way that can promote ice nucleation. Although the inductive effects of the solid surface of different materials are diverse and different in strength, it can be summarized that the type and structure of an IML depend on the competitive equilibrium of multiple inductive effects and the influence of guest molecules. In the next tensile test, we also leverage this obvious structural difference to further show its profound impact on the adhesion strength between solid surfaces.

### Adhesion Strength

**Figure 5:**
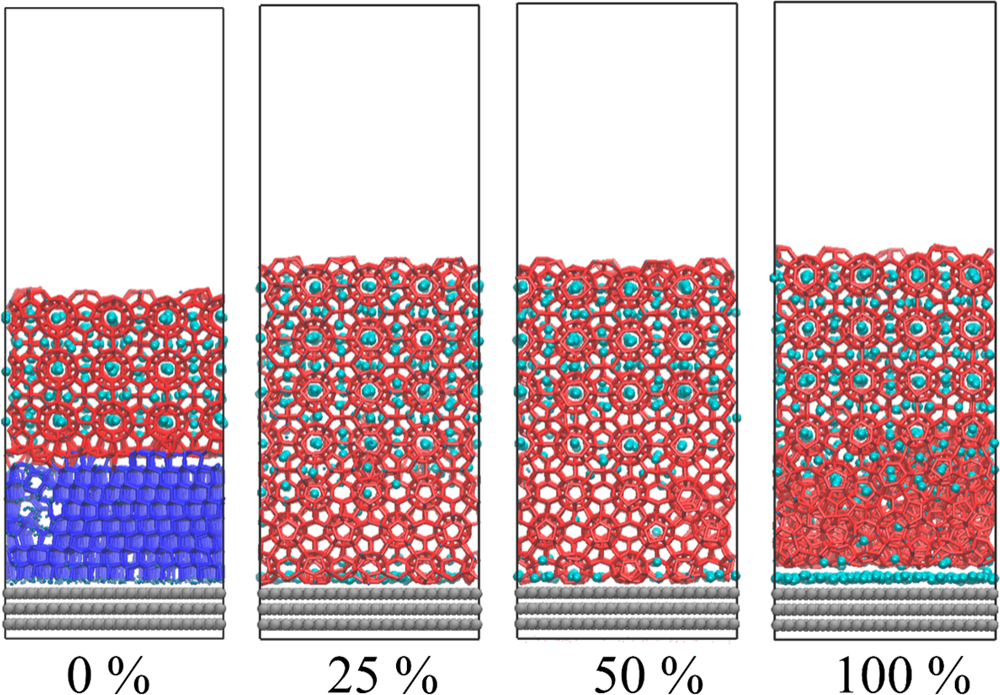
Configuration for tensile test. All is the final configuration after 100 ns growth in the previous MD process. The solid surface is shown with gray balls, guest molecules with cyan balls, liquid water with blue dots, and ice and hydrate with navy blue and red sticks, respectively.

There have been multiple studies focusing on the mechanical properties of bulk ice, hydrate, and their interfaces as well as the relevant adhesion mechanics.^49−53^ In the following part of this article, we focus on the adhesion strength of the prebuilt hydrate with IML on the solid surface. The tested model is the final configuration after 100 ns growth in the previous MD process, as shown in Figure 5. The IML of the model marked “0%” is an ice structure, and there is an obvious interface between different phases. The IML structure in the system labeled “25%” and “50%” are close to sI hydrate crystals, while closer to amorphous in the “100%” system. It is worth noting that there is an adsorption layer formed by guest molecules on the solid surface, that is, phase segregation. All tensile tests were performed by applying pulling force on the prebuilt hydrate crystals vertically away from the fixed solid surface to test the adhesion strength of the IML. All models use a uniform pulling velocity of 0.001 Å/fs, and another set of results are provided in SI as a control study with loading rate (0.0001 Å/fs) (SI Figure S5). Although the loading rate has a certain degree of influence on the absolute value of adhesion, the relative strength of adhesion based on the same loading rate is comparable.^54^

**Figure 6:**
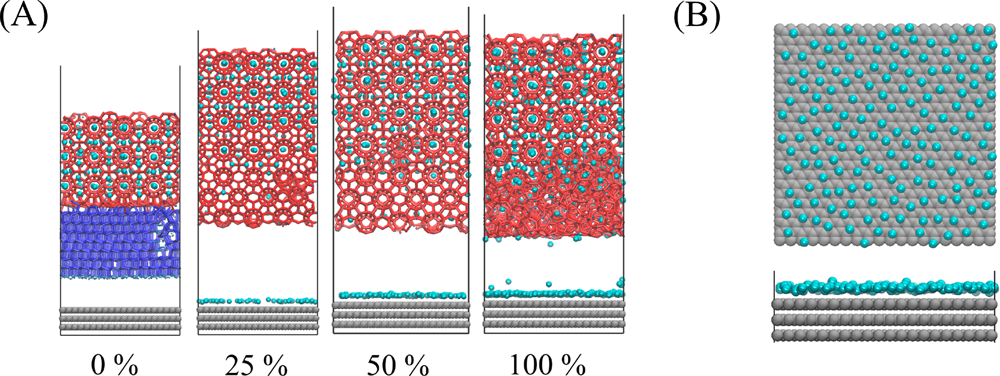
Fracture structure and gas adsorption on solid surface. (A) Snapshots of the IML fracture instant. The solid surface is shown with gray balls, guest molecules with cyan balls, liquid water with blue dots, and ice and hydrate with navy blue and red sticks, respectively. (B) A typical surface guest molecule adsorption layer.

Figure 6(A) shows the snapshots of the IML fracture instants. For all models with guest molecules, structural fracture initiates near the solid surface, namely the IML with the prebuilt hydrate together is detached from the solid surface. In contrast, fracture can occur either near the solid surface or, in rare cases, at the interface between the ice and the hydrate structure if the IML is ice. For most configurations with guest molecules, all water molecules are pulled away from the solid surface with the hydrate structure, leaving only part of the guest molecules on the solid surface. Figure 6(B) shows the configuration of a typical surface guest molecule adsorption layer. This can be largely attributed to the stronger interaction between water molecules through hydrogen bonds than the van der Waals force between the solid surface and water molecules. Therefore, along the stretching direction, the weakest position of the system almost appears at the interface between the IML and the solid surface. Given the nature of the surface model, this result is largely applied to such smooth and hydrophobic solid surfaces. In realistic conditions, there are other key surface properties, such as chemical composition, roughness and morphology, that could lead to varied hydrate detaching behaviors. The effect of different surface properties on hydrate detachment is subjected to future studies.

**Figure 7:**
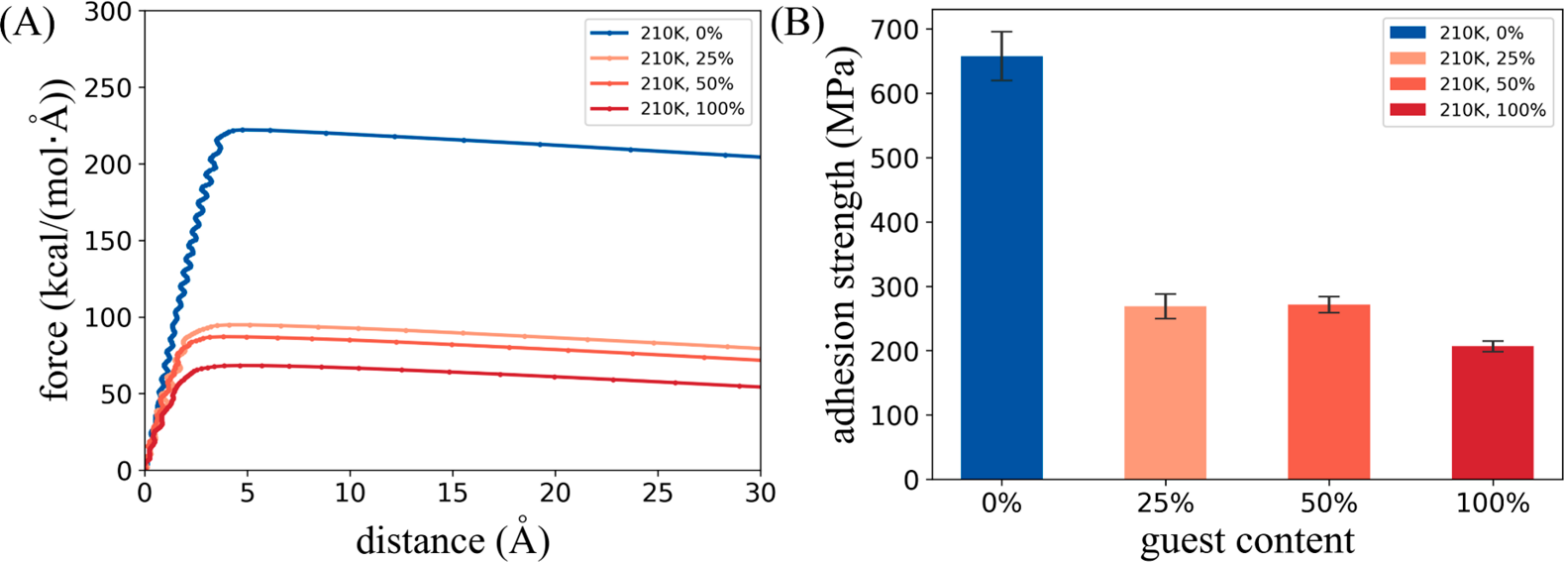
Force varies during the tensile test and adhesion strength. (A) Displacement of the prebuilt hydrate structure and monitored force curves during the stretching process for four models (#Run 1 as example). (B) Adhesion strength of different IML on solid surface.

The relationship between displacement of the prebuilt hydrate structure and the monitored force during the tensile tests is shown in Figure 7(A). It is interesting to see that the force required to “peel” the ice from the solid surface is much larger than that for the hydrate structure. This is consistent with the experimental phenomenon that the hydrate adhesion force is further lower than ice in the deicing and dehydrate experiments.^27^Figure 7(B) shows the adhesion strength of different IML structures. The adhesion strength of the hydrate-like IML shows a decreasing trend with the increase of concentration of guest molecules in the IML.

**Figure 8:**
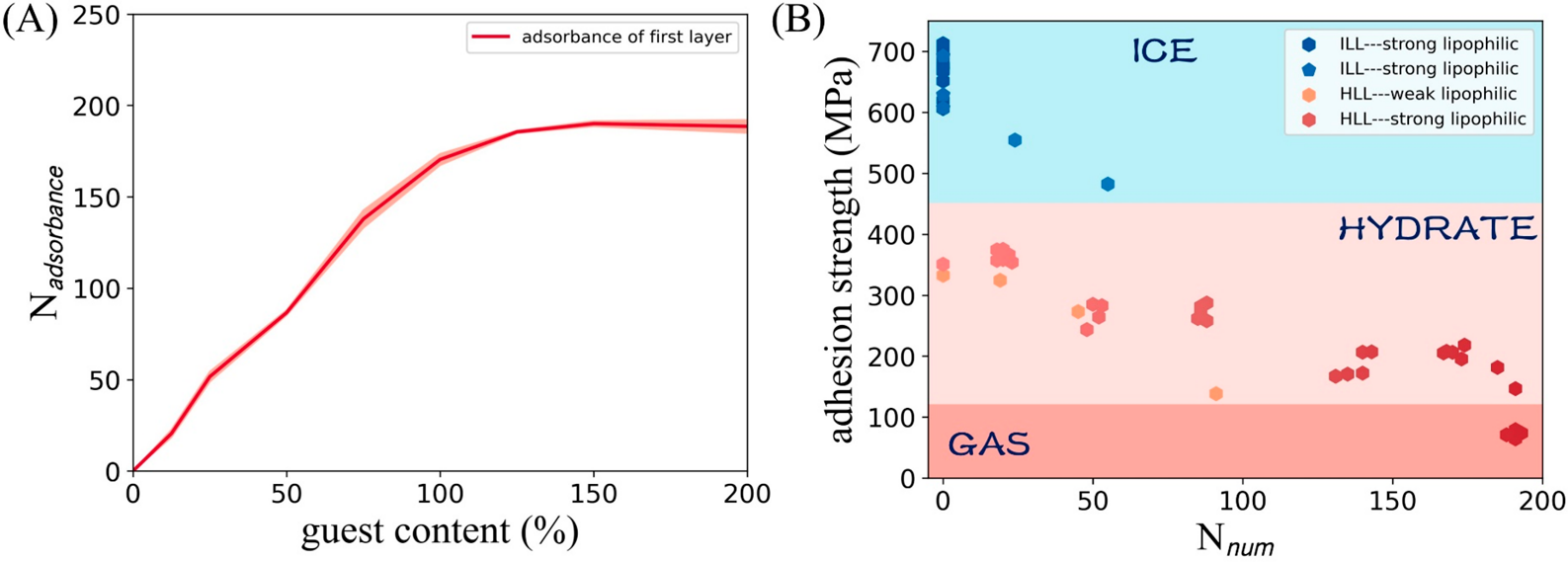
Effect of interfacial structure on adhesion strength. (A) The adsorption of the first adsorption layer changes with guest content in the system. (B) The relationship among IML structure, guest adsorbance on solid surface, and the adhesion value. The blue points series indicates the ice-like lattice (ILL) structure sample, and the red point series indicates a hydrate-like lattice (HLL) structure. Except for the orange points that are measured on a weak lipophilic surface (relatively more hydrophilic), the rest are obtained on a strong lipophilic surface. Hexagonal points indicate that the fracture site occurs abut on solid surface, while pentagonal points indicate that the fracture occurs at the hydrate–ice interface.

In order to further explore the influence of the guest adsorbance on solid surface on the adhesion strength of the IML, models with more guest molecules were built and subjected to the same simulation procedure and tensile testing. With the further increase of the content of guest molecules in the model, the adsorption of the first adsorption layer of guest molecules on the solid surface gradually increases and becomes saturated, as shown in Figure 8(A). Furthermore, the relationship among IML structure, guest adsorbance on solid surface, and the adhesion value are graphed and analyzed, as shown in Figure 8(B). Through the difference analysis for the trajectories represented by the outliers in the two sets of data (the adsorbed guest number ∼25 and ∼50), it is found that the formation of ice-like structure on solid surface is the reason causing the adhesion strength outlier. This observation shows that under the same adsorbance, the transformation of the lattice structure of water molecules will alter the adhesion. SI Figure S6 shows these two typical snapshots of the interface structure, which show the huge difference in areal density caused by the different structures of water molecules when the same guest molecule is adsorbed. In addition, the increase in the adsorption of guest molecules at the solid interface is another reason for further weakening the adhesion of the IML. Although the results of the simulations and experiments are not completely consistent in terms of the absolute value of adhesion, a ratio of 5/1 for the adhesion strengths between the ice and hydrate is surprisingly consistent when the guest monolayer adsorption is established (∼140 guests adsorbed).^27^ We can attribute the huge difference in the adhesion strength between the hydrate and ice structure to the joint contribution of the lattice change and the adsorption of guest molecules. By sampling the weak-affinity surface (ε_si_ = 0.09 kcal·mol^–1^),^37^ the influence of the wettability of the solid surface is reflected to a considerable extent. The main effect for adhesion strength of lower lipophilicity is limited in causing fewer guest molecules to adsorb onto the solid surface, and at the same time weaken the adhesion force between the two. The snapshots of these models and a typical density distribution are provided in SI Figures S7 and S8. When more adsorption layers are established, the interaction between the IML and the solid surface can even be completely ignored. At this time, the IML/solid interface will be completely transformed into IML/gas interface, see SI Figure S9. Therefore, the limiting range for the marked difference in adhesion strength can be specified, that is, is between the ice–solid adhesion strength and the IML/gas interface adhesion strength.

**Figure 9:**
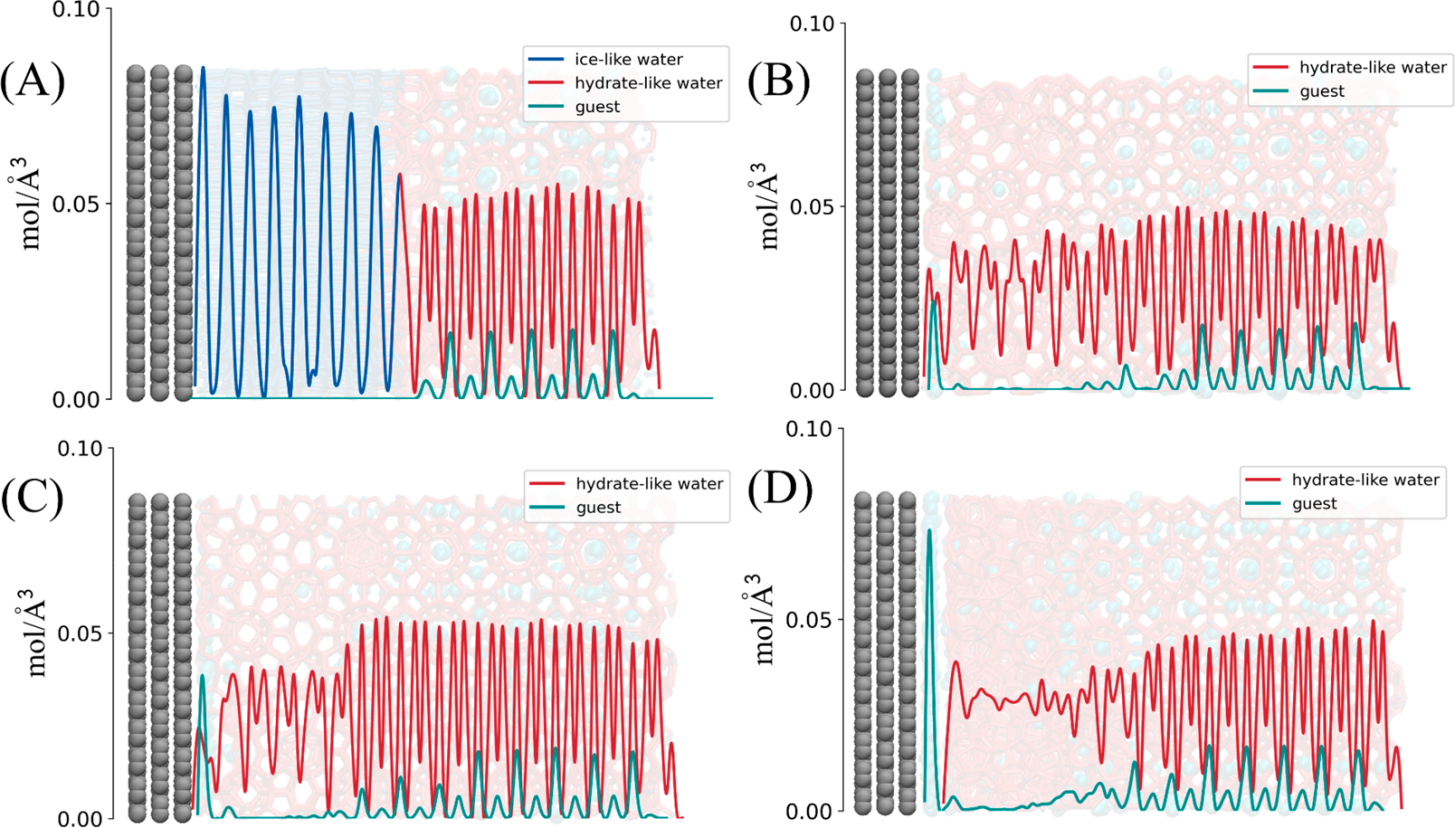
Density distribution of water molecules and guest molecules along the Z direction. (A) With 0% guest molecule content. Ice formation is the usual result for this system. (B) With 25% guest molecule content. The sI hydrate-like IML is the usual result for this system. (C) With 50% guest molecule content. The sI hydrate-like IML is the usual result for this system. (D) With 100% guest molecule content. Amorphous hydrate structures appear in systems with this or higher concentration of guest molecules.

From the density distribution of solid surface normal direction shown in Figure 9, it can be seen that the hydrate lattice and the ice lattice are significantly different. Although the bulk density of hydrate and ice is very similar, the hydrate structure “trades” more layers for lower surface density. From a molecular perspective, this low areal density reduces its adhesion on solid surface; it can also be used to explain the aforementioned few examples where the fracture position appears at the ice–hydrate interface. Taking the adhesion strength (Figure 8(B)) into account, it should be mentioned that the densely packed interface structure of the hexagonal ice leads to a significantly higher adhesion strength than that of others. As such, the areal water density adjacent to solid surface is another crucial factor to hydrate adhesion besides the known surface hydrophobicity identified by previous experiments.^24−27^ The results thus deepened the understanding of hydrate adhesion at the nanoscale. Moreover, with the adsorption of guest molecules, the water molecules at the interface are gradually replaced by guest molecules, and the water–solid interface is replaced by the water–gas interface, which further weakens the adhesion of hydrates (in actual situations, the interface caused by the local large bubbles can also be classified as this case). It is obvious that the nearest molecular layer has a great impact on hydrate/ice adhesion (Figures 8 and 9). Pure water nearest molecular layer led to the highest adhesion strength. Mixed water and gas in the nearest layer can result in reduced adhesion strength. Overall, the nearest layer of pure gas yields the lowest hydrate adhesion among the four systems. It is worth noting that adhesion strength of a similar range was observed in the systems with 25% and 50% of gas content, owing to the fact that there are no significant differences in the nearest molecular layers in the two systems (Figure 9B,C). Replacing a limited number of water molecules with gas molecules did not lead to a significant reduction in hydrate adhesion. Based on the above analysis, it is still an effective approach that reduces the adsorbance of water molecules and adsorbs or produces some weak adhesion species like the guest layer at the solid interface to weak hydrate adhesion, just like the strategy we adopted in the previous anti-icing work.^55,56^

## Conclusions

In this paper, molecular dynamics simulations were performed to study the formation process of the IML between the hydrate and the solid surface, and the corresponding adhesion strength of these different IML structures was obtained through tensile testing. Our results show that the ability to induce IML growth from both solid surface and hydrate together with the steric hindrance and hydrophobic effect of the guest molecules determine its structures. By comparing the mechanical performance of all systems, it is found that the areal density of the water molecular structure and amount of guest molecules’ adsorbance along the fracture plane are the dominant factors that determine the adhesion of hydrates. The difference in the crystal structure and component of the intermediate layer gives the theoretical interval of the hydrate adhesion strength. In addition, it is interesting that our simulation results show a ratio consistent with experimental reports:^27^ hydrate adhesion on a solid surface is approximately ^1^/_5_ of that of ice adhesion. Furthermore, it also implies that the hydrophobicity and ability of templated low-density water structuring are preferable properties for low hydrate adhesion surfaces. These results point toward the establishment of surface-engineering “design rules” to exploit, manipulate and optimize these molecular-templating phenomena in an effort to realize lower- or higher-adhesion surfaces for hydrate formation, depending on whether the motivation lies in the surface-mediated inhibition or promotion of hydrate growth for disparate industrial applications.

## Supporting Information Available

Ice growing in the IML, system stability, loading rate, Interface structure, weak-affinity surface, configuration of multilayer guest adsorption (PDF)
