# Supplementary material for: Unraveling Adhesion Strength between Gas Hydrate and Solid Surfaces
Source: Langmuir. 2021 Nov 16;37(47):13873–81. doi: 10.1021/acs.langmuir.1c02315 (PMC8638257; doi:10.1021/acs.langmuir.1c02315)
Supplement: Supplementary file 1 — la1c02315_si_001.pdf [file la1c02315_si_001.pdf]

Unraveling adhesion strength between gas hydrate and solid surfaces

Rui Ma[†], Feng Wang[†], Yuanhao Chang[†], Senbo Xiao [†] *, Niall English[‡], Jianying He[†], Zhiliang Zhang[†] *

[†]*NTNU Nanomechanical Lab, Department of Structural Engineering, Norwegian University of Science and Technology (NTNU), Trondheim 7491, Norway*

[‡]*School of Chemical and Bioprocess Engineering, University College Dublin, Dublin 4, Ireland*

The algorithm based on the local water network structure[1] and the CHILL+ algorithm[2] are used to identify the crystal structure in this work.

A. Ice growing in the IML

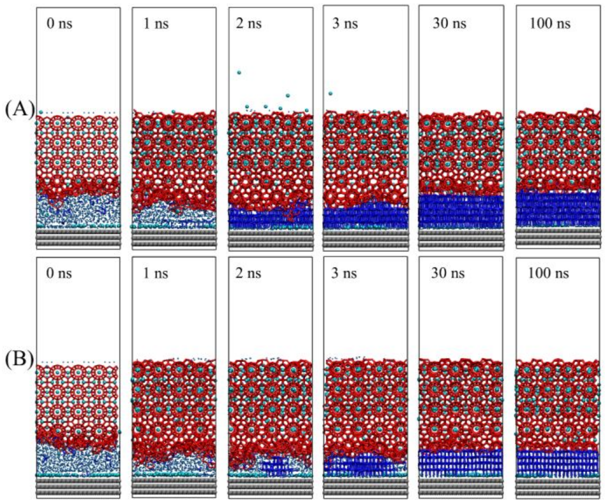

Figure S1. Snapshots of special IML with ice in systems containing guest molecules, and the adhesion rises sharply due to the ice structure appear on the solid surface (A) With 12% guest molecule content. (B) With 25% guest molecule content. The solid surface is shown with gray balls, guest molecules with cyan balls, liquid water with blue dots, and ice and hydrate with navy blue and red sticks respectively.

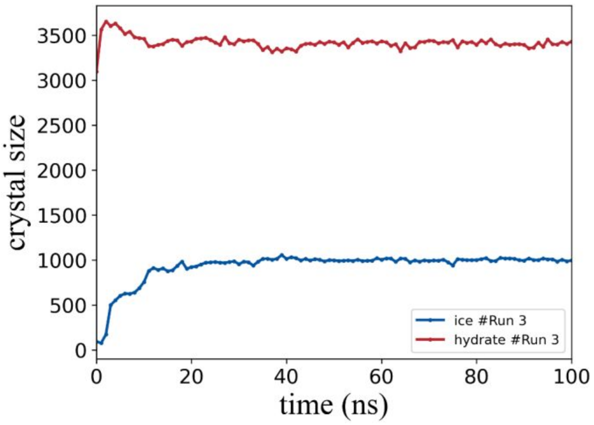

Figure S2. The growth curve of hydrate and ice in a "25%" model with ice structure. In this model, the adsorbed guest molecules are not able to prevent the nucleation and growth of ice.

B. System stability

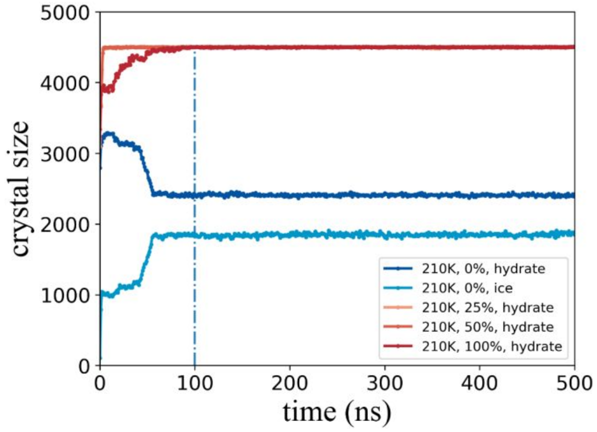

Figure S3. The growth curve of ice and hydrate structure in the system is used to describe the stability of the system structure. The simulation time is extended to 500 ns, and the structure of each system is stable after 100ns simulation time. In addition, the "25%" model and the "50%" model have very close growth curves of hydrate.

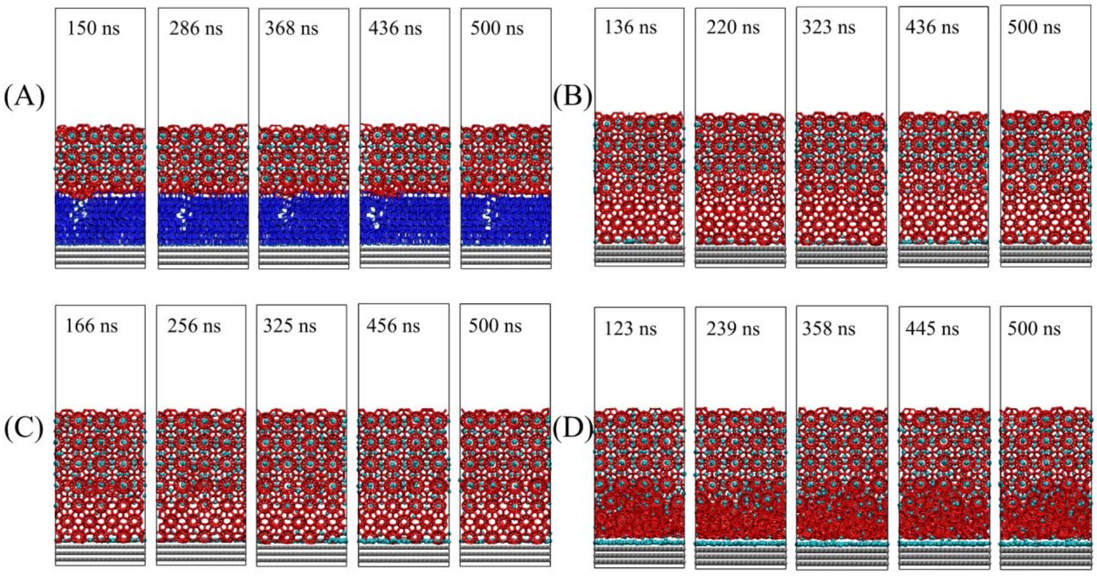

Figure S4. Snapshots of 100-500 ns simulations randomly selected in the systems. (A) With 0% guest molecule content. (B) With 25% guest molecule content. (C) With 50% guest molecule content. (D) With 100% guest molecule content. The solid surface is shown with gray balls, guest molecules with cyan balls, liquid water with blue dots, and ice and hydrate with navy blue and red sticks respectively.

C. Loading rate

Although the loading rate causes changes in the absolute magnitude of the adhesive force measured during the detachment process[3], the difference in the adhesive force of ice and hydrate on the surface is significant. In addition, since our results are based on the same loading rate for comparison, the obtained adhesion strength can be compared.

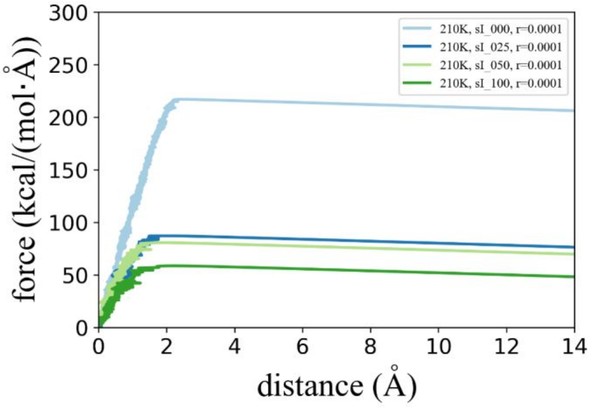

Figure S5. The displacement of the pulled pre-built hydrate structure and the monitored force curves of the four models during the detaching process when the loading rate r=0.0001.

D. Interface structure

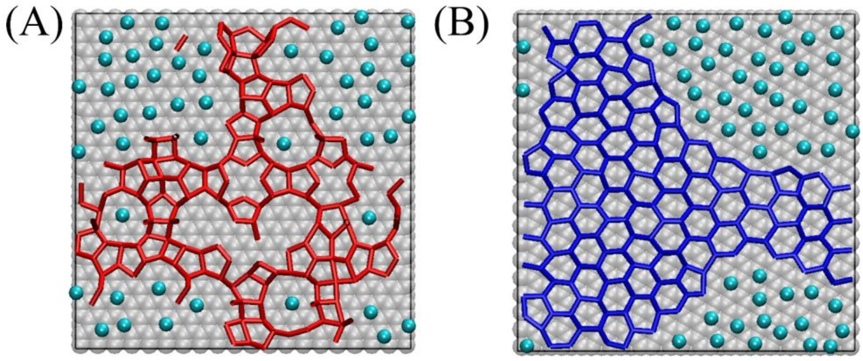

Figure S6. There is a significant difference in the areal density of water molecules in the different interface structure. The two configurations have almost the same amount of guest molecule adsorption. The solid surface is shown with gray balls, guest molecules with cyan balls, liquid water with blue dots, and ice and hydrate with navy blue and red sticks respectively.

E. Weak-affinity surface

The weak interaction strength (ε_{si} = 0.09 kcal·mol^{-1})[4] between solid surface and guest molecules results in easier desorbing of guest molecules and thus a decrease of guest adsorbance. Consequently, water molecules are more difficult to be replaced, which relatively reflect the effect of a more hydrophilic surface. These changes affect the distribution of guest molecules in the system, and “bubble-like” guest clusters are adsorbed on the solid surface, as the final effects shown by the snapshots in Figure S7. The density distribution along the normal direction of the solid surface in a typical system is shown in Figure S8. Due to the clustering of the guest molecules into “nano bubble”, direct contacting areas between water and solid surface emerge and maintain stably during simulation. Nevertheless, the concentration of guest molecules adsorbed on a solid surface is greatly reduced compared to a system with stronger lipophilic surface. And because of the smaller guest-surface interaction, its adhesion strength is significantly reduced.

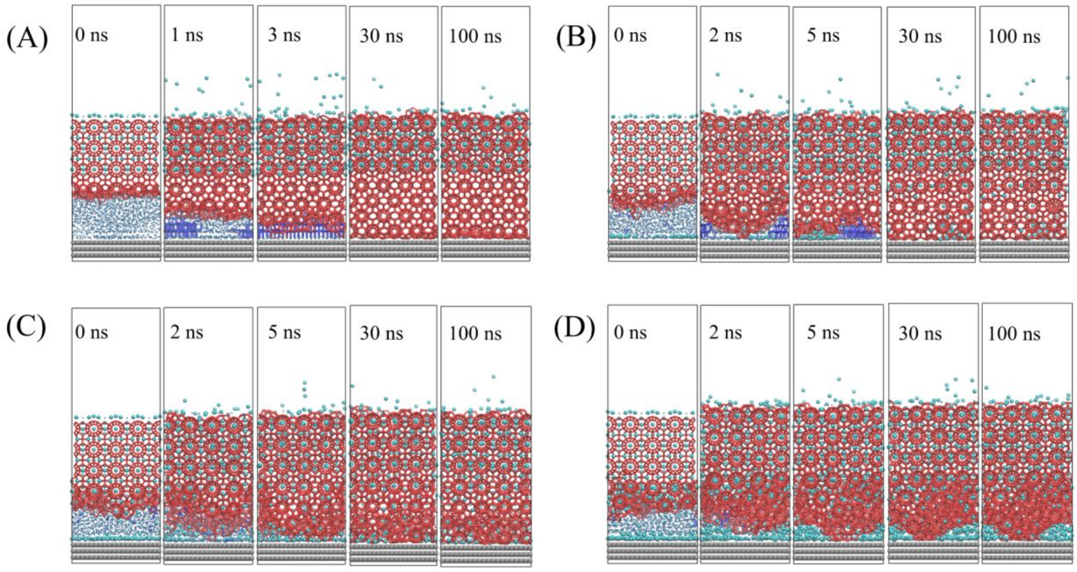

Figure S7. Snapshots of different MD systems with weak-affinity surface. (A) With 0% guest molecule content. (B) With 25% guest molecule content. (C) With 50% guest molecule content. (D) With 100% guest molecule content. The solid surface is shown with gray balls, guest molecules with cyan balls, liquid water with blue dots, and ice and hydrate with navy blue and red sticks respectively.

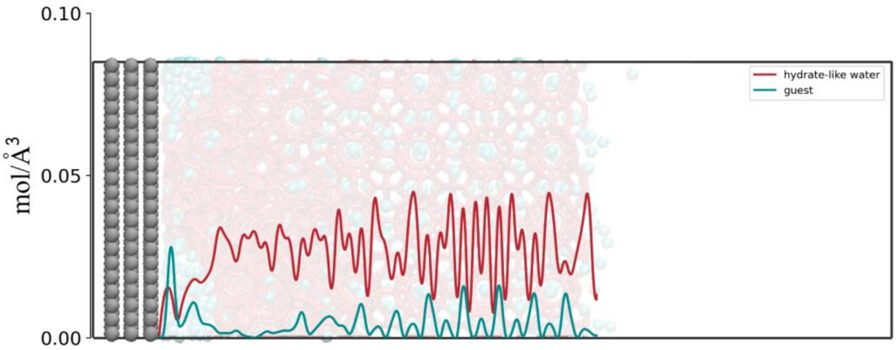

Figure S8. The density distribution of water molecules and guest molecules along the Z direction in system with weak-affinity surface. This system corresponds to the system where the concentration of guest molecules is marked as "100%".

F. Configuration of multilayer guest adsorption

With the continuous increase of the content of guest molecules in the intermediate layer, a multilayer adsorption of guest molecules is formed on the solid surface. The water structure-solid interface is transformed into a water structure-gas interface. The completion of this transformation determines the lower limit of the adhesion of the water structure.

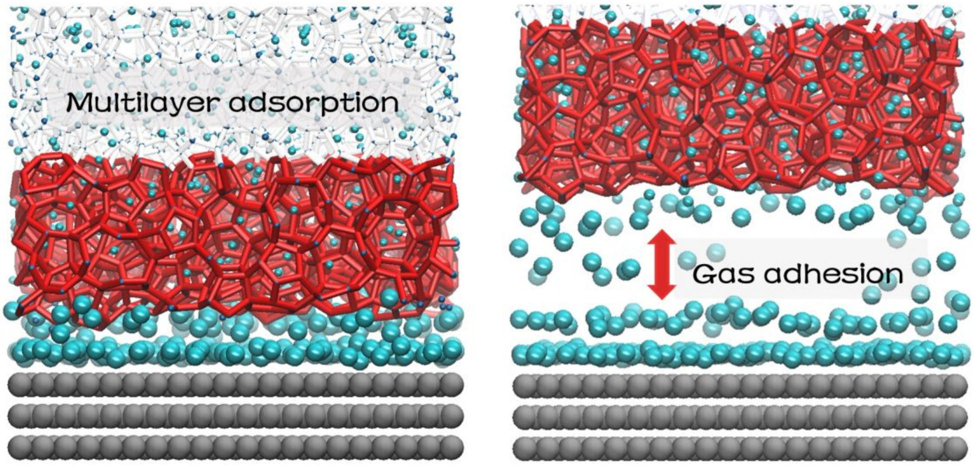

Figure S9. The fracture on multilayer adsorption layer. The solid surface is shown with gray balls, guest molecules with cyan balls, liquid water with blue dots, and hydrate with red or white sticks.

REFERENCES

1. Li, L.; Zhong, J.; Yan, Y.; Zhang, J.; Zeng, X. C., Unraveling nucleation pathway in methane clathrate formation. *Proceedings of the National Academy of Sciences* **2020,** *117* (40), 24701-24708.
2. Nguyen, A. H.; Molinero, V., Identification of clathrate hydrates, hexagonal ice, cubic ice, and liquid water in simulations: The CHILL+ algorithm. *The Journal of Physical Chemistry B* **2015,** *119* (29), 9369-9376.
3. Wu, J.; Ning, F.; Trinh, T. T.; Kjelstrup, S.; Vlugt, T. J.; He, J.; Skallerud, B. H.; Zhang, Z., Mechanical instability of monocrystalline and polycrystalline methane hydrates. *Nature communications* **2015,** *6* (1), 1-10.
4. Ma, R.; Zhong, H.; Li, L.; Zhong, J.; Yan, Y.; Zhang, J.; Liu, J., Molecular Insights into the Effect of a Solid Surface on the Stability of a Hydrate Nucleus. *The Journal of Physical Chemistry C* **2020,** *124* (4), 2664-2671.
